# The spleen size in patients undergoing hemodialysis

**DOI:** 10.1590/2175-8239-JBN-2020-0116

**Published:** 2020-10-19

**Authors:** Nordeval Cavalcante Araújo, José Hermógenes Rocco Suassuna

**Affiliations:** 1 Universidade do Estado do Rio de Janeiro, Departamento de Nefrologia, Rio de Janeiro, RJ, Brasil.

**Keywords:** Renal Dialysis, Blood Platelets, Polysomnography, Splenomegaly, Diálise Renal, Plaquetas, Ultrassonografia, Esplenomegalia

## Abstract

**Introduction:**

Inflammation promotes the progression of chronic renal failure, and the start of dialysis worsens inflammation. The enlargement of the spleen is associated with inflammation, and patients on hemodialysis may show a large spleen. The aim of the present study was to compare the spleen size of patients undergoing hemodialysis versus controls to update this thread.

**Methods:**

Controls and patients were eligible to participate in the study provided they were negative for serological markers of hepatitis B and C viruses and HIV, if they had no lymphoproliferative disorder, and if they were at least 18 years of age. Age, sex, and the duration of dialysis were recorded. Laboratory variables (hemoglobin, hematological cell count, serum creatinine) and the underlying cause of end-stage renal disease were analyzed. The spleen sizes of the patients were divided into tertiles.

**Results:**

The 75 controls and 168 patients selected were sex-matched. The patients were older, had larger spleens and lower platelet counts than controls. The relationship between spleen size and age in the controls and patients was quite similar. The patients in the first tertile of spleen size compared with those in the third were older and had a higher platelet counts. The underlying disease and dialysis vintage had no effect on spleen size.

**Discussion:**

The patients had larger spleens and a greater range of spleen sizes than the controls. In patients, the association between larger and smaller spleen with lower and higher platelet counts, respectively, sparked the speculation of occurrence of hypersplenism and hyposplenism.

## Resumo

**Introdução:**

A inflamação promove a progressão da insuficiência renal crônica, e o início da diálise agrava a inflamação. O aumento do baço está associado à inflamação e os pacientes em hemodiálise podem apresentar um baço grande. O objetivo do presente estudo foi comparar o tamanho do baço de pacientes em hemodiálise versus aquele de controles, para atualizar este tópico.

**Métodos:**

Controles e pacientes foram elegíveis para participar do estudo desde que fossem negativos para marcadores sorológicos dos vírus da hepatite B, C e HIV, se não apresentassem distúrbio linfoproliferativo e tivessem pelo menos 18 anos de idade. Registramos idade, sexo e duração da diálise. Avaliamos as variáveis laboratoriais (hemoglobina, contagem de células hematológicas, creatinina sérica) e a causa básica da doença renal terminal. O tamanho dos baços dos pacientes foram divididos em tercis.

**Resultados:**

Os 75 controles e 168 pacientes selecionados foram pareados por sexo. Os pacientes eram mais velhos, tinham baços maiores e menor contagem de plaquetas do que os controles. A relação entre o tamanho do baço e a idade dos controles e pacientes foi bastante semelhante. Os pacientes do primeiro tercil de tamanho do baço, em comparação com os do terceiro, eram mais velhos e apresentavam contagens de plaquetas mais altas. A doença subjacente e o período de diálise não tiveram efeito no tamanho do baço.

**Discussão:**

Os pacientes tinham baços maiores e uma maior variedade de tamanhos de baço do que os controles. Entre os pacientes, a associação entre baço maior e menor com contagens de plaquetas mais baixas e mais altas, respectivamente, gerou a especulação da ocorrência de hiperesplenismo e hiposplenismo.

## Introduction

Inflammation has a role in the progression of chronic renal failure^1^, regardless of the etiology. In end-stage renal disease, the start of dialysis treatment acts as a triggering event that worsens inflammation^2^. In addition, repeated dialysis treatments lead to leucocyte activation and, consequently, the production of cytokines^3^. The persistence of inflammation contributes to the overall and cardiovascular mortality associated with this condition^4^.

The pathophysiology of inflammation includes recruitment of leucocytes to the spleen^5^, and enlargement of the organ has been reported to be associated with inflammation^6^. According to publications from the 1970s and early 1980s, patients undergoing hemodialysis showed enlargement of the spleen^7^^-^ 9. In line with this latter finding, it is well-known that hypersplenism may appear in patients undergoing regular hemodialysis^10^. On the other hand, hyposplenism has also been reported to be associated with some conditions that cause end-stage renal disease (ESRD)^11^^-^ 12. Moreover, the association between hyposplenism and renal transplantation has been established based on Howell-Jolly bodies in blood smears^13^ and hepatosplenic scintigraphy^14^. However, it has not been determined if hyposplenism develops after transplantation or if the condition is already present before starting immunosuppression.

The aim of the present study was to shed light on this issue by comparing the spleen size in patients undergoing hemodialysis versus a control group and by analyzing the determinant factors related to the length of the spleen to update this thread.

## Materials and Methods

This was a cross-sectional study carried out in the nephrology facility of the Hospital Pedro Ernesto of the University of the State of Rio de Janeiro. All kidney donor candidates and ESRD patients undergoing renal replacement therapy referred for sonography in the period from 2008 to 2019 were eligible to participate in the study. Since 2010, most sonography examination at our facility have involved evaluation of the kidneys and spleen. In accordance with the aim of the study, only cases in which kidney and spleen were scanned at the same time were enrolled in the study. The inclusion criteria were as follows: (1) negative serological markers of hepatitis B (HBsAg) and hepatitis C (anti-HCV) viruses and human immunodeficiency virus (anti-HIV), (2) no lymphoproliferative disorder, and (3) at least 18 years of age. Patients on peritoneal dialysis and those referred for hemodialysis under immunosuppressive treatment because of early or late renal transplant dysfunction were excluded.

In addition to age and sex, the duration of dialysis treatment was also recorded. Laboratory variables (hemoglobin, hematological cell count, and serum creatinine) and the underlying cause of ESRD were obtained from medical records of the patients. *A platelet count of* <150,000/mm^3^ was used as the threshold value *for diagnosis of thrombocytopenia^15^,* while a platelet count higher than 450,000/mm^3^ was indicated as thrombocytosis^16^.

The protocol for spleen sonography has already been reported elsewhere^13^. In brief, different angles of insonation at different sites were performed in order to display the best image, in which the most cranial and most caudal edge of the spleen were seen in the scan plane for measuring spleen length. All ultrasound studies were performed by the same observer (NCA).

## Statistical Analysis

The normality of data distribution was assessed by means of the Wilks-Shapiro test. In accordance with normality of data, the continuous variables studied in the controls and patients on hemodialysis were compared using parametric (Student's t-test) or non-parametric tests (Mann-Whitney test). The strength of the relationship between continuous variables was evaluated using Pearson's correlation coefficient. The associations between categorical variables were evaluated using the Chi-square test. The spleen size values of the study group were divided into tertiles. The subsets (tertiles) were defined by spleen size cut-off values reflecting the 33rd and 66th percentiles of spleen size distribution. The statistical significance threshold was set at p<0.05.

The institutional ethics committee approved the study protocol with the waiver for informed consent due to the retrospective nature of the study.

## Results

From May 2010 until February 2020, out of 3009 sonograms performed in 1281 different subjects, spleen sonograms were available for 1070 subjects. Of these, 763 patients were excluded because they were transplanted (440 patients), were on conservative treatment (297 patients), or were on continuous ambulatorial peritoneal dialysis (26 patients).

Seventy-five subjects with normal renal function, including renal donor candidates, patients with minor renal abnormalities (microscopic hematuria, kidney stone patients without obstruction, non-nephrotic proteinuria), and patients referred for routine health check-up, were assigned to the control group. Of the 232 patients undergoing hemodialysis, 62 were ruled out because serological tests for hepatitis B or C or HIV were positive or not available, and 2 were ruled out because they were under 18 years of age. After exclusion of these patients, 168 remained eligible for the study group.

The main underlying etiologies of ESRD were diabetes mellitus (41; 24.4%), hypertension (40; 23.8%), glomerulopathy (27; 16.1%), polycystic kidney disease (8; 4.8%), unknown causes (35; 20.8%), and miscellaneous causes (17; 10.1%). Patients with concomitant diabetes and hypertension were assigned to diabetes group, and patients with lupus erythematosus were assigned to glomerulopathy group. Both groups had similar patterns of sex distribution (Chi-square =0.005; p=0.942). Table 1 shows the continuous variables studied and the statistical differences between the controls and hemodialysis patients. Patients undergoing hemodialysis were older (p<0.014), had larger spleens (p<0.001), lower hemoglobin levels (p<0.001), higher leukocyte (p=0.021), neutrophil (p<0.001), and monocyte (p<0.001) counts, and lower lymphocyte (p<0.001) and platelet counts (p=0.001) than controls (Table 1). Using a threshold value of <150,000/mm^3^ or >450,000/mm^3^ for platelet count, the present study showed a 16.0% incidence of thrombocytopenia in hemodialysis patients and a 1.9% incidence of thrombocytopenia in the control group (Chi-square = 7.233; p=0.007), while there was 0.6% incidence of thrombocytosis in hemodialysis patients and 0.0% incidence of thrombocytosis in the control group (Chi-square = 0.327; p=0.755).

**Table 1:** Comparison between control group and end-stage renal disease (ESRD) patients on hemodialysis

Variable	Controls, n=75	ESRD, n=168	p
Sex, male*	45.3 %	45.8 %	0.942
Age, years	44.87±14.75	49.89±16.79	0.017
Spleen size, mm	96.55±16.03	106.41±17.07	<0.001
Hematocrit, %	41.16±4.05	29.09±7.79	<0.001
Hemoglobin, g/dL	13.64±1.47	9.39±2.54	<0.001
Leucocytes, cells/mm^3^	7113±2141	8184±3485	0.049
Neutrophils, cells/mm^3^	4184±1831	5612±3135	<0.001
Lymphocytes, cells/mm^3^	2217±725	1654±789	<0.001
Monocytes, cells/mm^3^	478±177	637±342	0.001
Platelets, cells/mm^3^	260415±63210	222025±75281	<0.001
Creatinine, mg/dL	0.88±0.19	7.67±3.02	<0.001

Mann-Whitney test was used unless otherwise specified.

* Chi-Square test.

**Figure 1:**
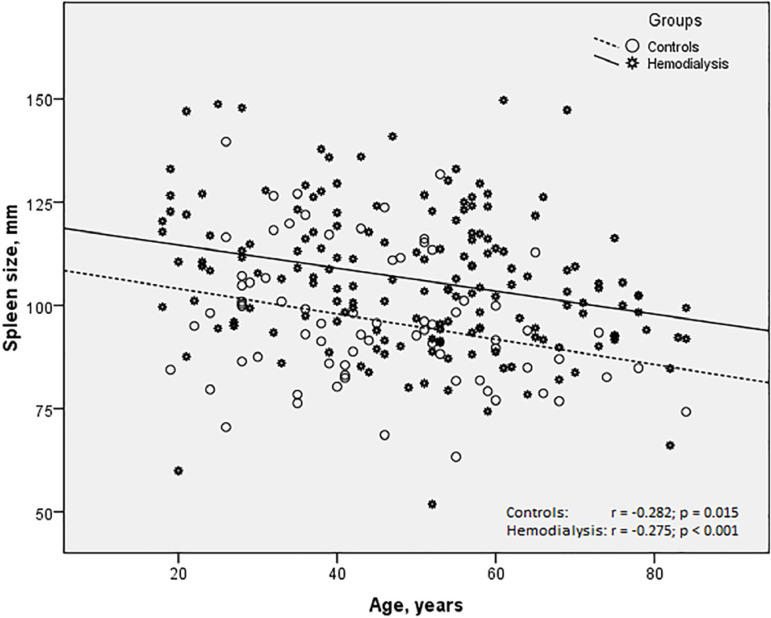
Correlation between spleen size and age in controls and end-stage renal disease patients undergoing hemodialysis.

There was a greater distribution of spleen size in the study group (minimum = 51.8 mm, maximum 149.7 mm, range = 97.9 mm) than in the controls (minimum = 63.3 mm, maximum 139.6 mm, range = 76.3 mm). The correlation coefficient between spleen size and age in the controls and patients on hemodialysis was quite similar (Figure 1). Among patients on hemodialysis, the comparison of the first and third tertile of spleen size revealed that patients with smaller spleens were predominantly women (p=0.029), older (p=0.004), had statistically significant higher platelet counts (p=0.023) (Table 2), and a lower incidence of thrombocytopenia (10.9% vs 25.9%; Chi-Square = 4.101; p=0.043), but the incidence of thrombocytosis was similar in both groups (1.8% vs 0.0%; Chi-Square = 0.991; Fisher's Exact Test = 0.505). No association was found between diabetes mellitus or glomerulopathy and the smallest or largest tertiles of spleen size (smallest: 81.0% vs 19.0%; largest: 66.7% vs. 33.3%; Chi-Square = 0.952; Fisher's Exact Test = 0.277) (Table 2).

**Table 2:** Comparison between first (smaller) and third (larger) tertiles of spleen size in end-stage renal disease (ESRD) patients undergoing hemodialysis

Variable	Smaller, n=58	Larger, n=55	p
Sex, male*	41.2 %	58.8 %	0.050
DM vs glomerulopathy*	81.0% vs 66.7%	19.0% vs. 33.3%	0.277
Age, years	55.67±15.63	43.31±15.32	<0.001
Dialysis vintage, mo	13.45±25.66	28.64±44.81	0.256
Spleen size, mm	88.77±8.95	125.43±9.76	<0.001
Hematocrit, %	28.42±6.62	29.94±9.12	0.617
Hemoglobin, g/dL	9.19±2.15	9.65±2.92	0.801
Leucocytes, cells/mm^3^	8319±3542	7596±3676	0.109
Neutrophils, cells/mm^3^	5601±3210	5123±3271	0.163
Lymphocytes, cells/mm^3^	1753±999	1638±703	0.559
Monocytes, cells/mm^3^	679±360	542±284	0.059
Platelets, cells/mm^3^	233891±69702	203463±78656	0.023
Creatinine, mg/dL	7.59±3.03	8.18±2.98	0.226

Mann-Whitney test was used unless otherwise specified.

* Chi-Square test. DM: diabetes mellitus; mo: months.

## Discussion

The most important contribution of this study is that it confirmed the finding of previously published reports about enlarged spleens in patients undergoing hemodialysis. Indeed, by means of sonography we were able to reproduce the results of studies carried out using the estimated volume by scintigraphy^9^ and weight at autopsy^8^ and after splenectomy^7^. Moreover, in this study, the use of a control group increased the reliability of the results (Table 1). It is well-known that men have larger spleens than women^17^. Therefore, it is worth mentioning that the two groups in this study were sex-matched. On the other hand, the difference in mean age of both groups deserves some comments. Although, in this study, the subjects in the control group were younger than the subjects in the hemodialysis group, the effect of age on spleen size was the opposite of what one would expect based on the results. That is, subjects in the hemodialysis group (older) should have smaller spleens than subjects in the control group (younger). Moreover, Figure 1 highlights this problem, showing that the relationship between age and spleen size in the controls and hemodialysis patients had no effect on the correlation coefficient (Figure 1). However, the imaginary straight line that describes the trajectory of the data of both correlations was almost parallel, and the line of the hemodialysis group was higher that that of the controls, indicating that for each age the spleen size is larger in this group. Therefore, the difference in spleen size in the controls and hemodialysis group could not be attributed to differences in sex or age distribution in both groups. If young patients on hemodialysis will develop age-related decreases in spleen size, despite continuing treatment, can only be addressed by means of a longitudinal study.

The effect of the underlying cause of ESRD was assessed by comparison of diabetes, a condition not mainly related to immune responses, with glomerulopathy, a group of conditions in which the immune system plays a key role in the pathophysiology of the disease. Moreover, in the latter case, the treatment includes many drugs that suppress the immune system. Since no association has been found between glomerulopathy or diabetes with spleen size, it is reasonable to wonder if the underlying disease or treatment before the start of hemodialysis has an effect on the spleen size of these patients. That is, the effect of ESRD on hemodialysis itself might be the cause of the enlargement of the spleen. In support of these thoughts, data from autopsies of patients with chronic glomerulonephritis showed that spleens were more than twice as heavy in patients on hemodialysis (173±15 g) than those not on hemodialysis (81±15 g)^8^.

In the past, splenomegaly has been attributed to venous congestion secondary to fluid overload, viral hepatitis, cirrhosis, or stimulation of the immune system by chronic bacterial or viral infections, or the presence of foreign particles in the pulp^9^. In the present study, viral infections were excluded. The patients were in outpatient hemodialysis without need of further treatment of fluid overload. Foreign particles in the spleen pulp are out of the scope of this study. Therefore, it is reasonable to speculate that the increased spleen may result from hemodialysis itself.

Based on this finding, it is appropriate to point out that despite development of new techniques to improve biocompatibility of medical polymers used in hemodialysis devices in recent decades, this study showed that the effect of hemodialysis on spleen size is the same as that reported in previous work. The lower platelet count and higher incidence of thrombocytopenia in patients than controls suggest increased splenic platelet destruction.

The dispersion of spleen size in the hemodialysis group was greater than that of the controls. As a consequence, the patients located in the extreme tails of the distribution showed smaller or larger spleens. These patients were included in the lowest and highest tertiles of spleen size, respectively. In comparison to the highest tertile, patients with a smaller spleen had statistically significant higher platelet counts (p=0.023) (Table 2), a finding that suggests that different spleen sizes have different effects on the removal of platelets. However, it was not possible to determine the underlying mechanism: small spleens associated with less sequestration or large spleens associated with more sequestration. The extreme values (small and large) of spleen size in the tails of the distribution might be the anatomical basis for the speculation about the occurrence of hyposplenism and hypersplenism in patients on hemodialysis.

In patients on hemodialysis, there are two theoretical consequences of hyposplenism we should keep in mind. First, the role of hyposplenism in arteriovenous fistula loss should not be neglected. Hyposplenism can be accompanied by thrombocytosis^18^^-^ 19, which might lead to increased risk of thromboembolic events^20^^-^ 21. *In post-splenectomy, the hypercoagulable state due to thrombocytosis probably contributes to the increased risk of fatal myocardial ischemia^22^. In functional hyposplenism, pathological findings are quite similar to those found in splenectomized patients^20^,^23^^-^24.* Based on this evidence, it is reasonable to speculate whether patients on hemodialysis with features of hyposplenism, are at the same risk of vascular access events or vascular access failure, as reported in abnormalities of mean platelet volume^25^^-^ 26. Second, the main adverse events of hyposplenic states are immunological and infectious events^21^. Hyposplenism might impair the antibody response to vaccination^27^^-^ 29, including hepatitis B vaccination^30^. In patients on regular hemodialysis, the overall primary vaccine-induced response to hepatitis B vaccination was impaired in a similar fashion^31^.

The occurrence of hypersplenism in hemodialyzed patients is well documented^10^^,^ 32. In cases of hypersplenism treated with splenectomy or partial embolization, the clinical features of hypersplenism were leucopenia or pancytopenia^10^^,^ 32. The reversion of the isolated thrombocytopenia to a normal count after splenectomy has also been reported^33^. Although heparin-induced thrombocytopenia has been claimed to be associated with hemodialysis, according to some studies, the decreased platelet count was similar to the alternative anticoagulant regimen^34^. Viral hepatitis (B or C) is another factor associated with thrombocytopenia in ESRD patients undergoing hemodialysis^35^^-^ 36. However, in our study, every patient received only heparin as an anticoagulant regimen, and patients with viral hepatitis (B or C) were excluded. Therefore, possible effects of heparin or viral hepatitis are unsuitable for explaining the cause of thrombocytopenia in a particular tertile of spleen size. However, it is reasonable to assume that heparin-induced thrombocytopenia could have partially blunted the development of thrombocytosis in patients with a small spleen.

In the past, pancytopenia with excessive transfusion requirements^10^ or a large spleen along with splenomegaly aroused suspicion of hypersplenism^37^. Today, in selected patients, this diagnosis should still be kept in mind. In the current study, patients with larger spleens had lower platelet counts, a hematological feature commonly found in hypersplenism.

Even though there is large evidence of spleen enlargement in hemodialyzed patients in the literature, it is believed that this concept may have no implications in clinical practice^38^.

The weakness of this study was the same as other cross-sectional studies, that is, no cause and effect can be determined. Moreover, although the main theoretical implications of small and large spleens (i.e., hyposplenism and hypersplenism) are suggested by platelet counts, none of these conditions has been further assessed with appropriated methods.

In conclusion, patients on hemodialysis have larger spleens than controls and a large dispersion of size. Patients with smaller and larger spleens were associated with higher and lower platelet counts, respectively. These findings raise speculation of the occurrence of hyposplenism and hypersplenism in this group of patients.

## References

[1] Silverstein DM (2008). Inflammation in chronic kidney disease: role in the progression of renal and cardiovascular disease. Pediatr Nephrol.

[2] Jofré R, Rodriguez-Benites P, López-Gómez JM, Pérez-Garcia R (2006). Inflammatory syndrome in patients on hemodialysis. J Am Soc Nephrol.

[3] Girndt M, Kaul H, Leitnaker CK, Sester M, Sester U, Köhler H (2001). Selective sequestration of cytokine-producing monocytes during hemodialysis treatment. Am J Kidney Dis.

[4] Taheri S, Baradaran A, Aliakbarian M, Mortazavi M (2017). Level of inflammatory factors in chronic hemodialysis patients with and without cardiovascular disease. J Res Med Sci.

[5] Li Y, Wu J, Xu L, Wu Q, Wan Z, Li L (2017). Regulation of leukocyte recruitment to the spleen and peritoneal cavity during pristane-induced inflammation. J Immunol Res.

[6] Mercier S, Breuillé D, Mosoni L, Obled C, Mirand PP (2002). Chronic inflammation alters protein metabolism in several organs of adult rats. J Nutr.

[7] George CR, Tremann JA, Quadracci LJ, Striker GE, Marchioro TL (1972). The spleen in chronic renal failure and renal transplantation. Proc Clin Dial Transplant Forum.

[8] Morohoschi T (1977). Enlargement of hemodialyzed spleen in uremia - histopathological and biometrical studies compared with the kidney. Acta Pathol Jpn.

[9] Platts MM, Anastassiades E, Sheriff S, Smith S, Bartolo DC (1984). Spleen size in chronic renal failure. Br Med J.

[10] Neiman RS, Bischel MD, Lukes RJ (1973). Hypersplenism in the uremic hemodialyzed patient: pathology and proposed pathophysiologic mechanisms. Am J Clin Pathol.

[11] Neilan BA, Berney SN (1983). Hyposplenism in systemic lupus erythematosus. J Rheumatol.

[12] McVicar MI, Chandra M, Margouleff D, Zanzi I (1986). Splenic hypofunction in the nephrotic syndrome of childhood. Am J Kidney Dis.

[13] Araújo NC, Lucena SBSG, Rioja SDS (2013). Functional hyposplenism in long-standing renal transplant recipients. Transplant Proc.

[14] Araújo NC, Neves MB, Mandarim-de-Lacerda CA, Orlando MMC (2017). Assessment of spleen filtrate function in renal transplant recipients using technetium-99m stannous colloid liver-spleen scan. Transplant Proc.

[15] Gauer RL, Braun MM (2012). Thrombocytopenia. Am Fam Physician.

[16] Schafer AI (2004). Thrombocytosis. N Engl J Med.

[17] Chow KU, Luxembourg B, Seifried E, Bonig H (2016). Spleen size is significantly influenced by body height and sex: establishment of normal values for spleen size at us with a cohort of 1200 healthy individuals. Radiology.

[18] Mohamed M (2014). Functional hyposplenism diagnosed by blood film examination. Blood.

[19] Subran B, Salama L, Dreyfus M, Carbonnel F, Besson C (2010). Thrombosis in acquired hyposplenism associated with Crohn disease. Presse Med.

[20] Kirkineska L, Perifanis V, Vasiliadis T (2014). Functional hyposplenism. Hippokratia.

[21] William BM, Corazza GR (2007). Hyposplenism: a comprehensive review. Part I: basic concepts and causes. Hematology.

[22] Robinette CD, Fraumeni JFF (1977). Splenectomy and subsequent mortality in veterans of the 1939-45 war. Lancet.

[23] Brigden ML (2001). Detection, education and management of the asplenic or hyposplenic patient. Am Fam Physician.

[24] Halfdanarson TR, Litzow MR, Murray JA (2007). Hematologic manifestations of celiac disease. Blood.

[25] Lano G, Sallée M, Pelletier M, Bataille S, Fraisse M, Berda-Haddad Y (2019). Mean platelet volume predicts vascular access events in hemodialysis patients. J Clin Med.

[26] Shin DH, Rhee SY, Jeon HJ, Park JY, Kang SW, Oh J (2017). An increase in mean platelet volume/platelet count ratio is associated with vascular access failure in hemodialysis patients. PLoS One.

[27] Schwartz AD, Pearson HA (1972). Impaired antibody response to intravenous immunization in sickle cell anemia. Pediatr Res.

[28] Hosea SW, Burch CG, Brown EJ, Berg RA, Frank MM (1981). Impaired immune response of splenectomised patients to polyvalent pneumococcal vaccine. Lancet.

[29] Di Padova F, Dürig M, Wadström J, Harder F (1983). Role of spleen in immune response to polyvalent pneumococcal vaccine. Br Med J.

[30] Kaddah N, Kaddah A, Omar N, Mostafa A (2010). Antibody response to hepatitis b immunization in Egyptian children with sickle cell disease. Egypt J Pediatr Allergy Immunol.

[31] Chaves SS, Daniels D, Cooper BW, Malo-Schlegel S, MacArthur S, Robbins KC (2011). Immunogenicity of hepatitis B vaccine among hemodialysis patients: effect of revaccination of non-responders and duration of protection. Vaccine.

[32] Spigos DG, Jonasson O, Mozes M, Capek V (1979). Partial splenic embolization in the treatment of hypersplenism. AJR Am J Roentgenol.

[33] Verzola A, Scapoli GL, Risichella IS, Prandini N, Rigolin M, Bergami M (2000). 'Isolated' thrombocytopenia by splenic sequestration in hemodialyzed patients. Nephron.

[34] Daugirdas JT, Bernardo AA (2012). Hemodialysis effect on platelet count and function and hemodialysis-associated thrombocytopenia. Kidney Int.

[35] Iwamoto Y, Ando M, Tsuchiya K, Nihei H (1999). Clinical analysis of thrombocytopenia in chronic dialysis patients. Jpn J Nephrol.

[36] Orasan OH, Urian L, Ciulei G, Breaban I, Stefan AM (2018). Thrombocytopenia in end-stage renal disease and chronic viral hepatitis B or C. J Mind Med Sci.

[37] Paganini EP, Garcia J, Abdulhadi M, Lathim D, Giesman J, Weick JK (1989). The anemia of chronic renal failure Overview and early erythropoietin experience. Cleve Clin J Med.

[38] National Kidney Foundation (2015). KDOQI clinical practice guideline for hemodialysis adequacy: 2015 update. Am J Kidney Dis.

