# Erratum

**DOI:** 10.1590/2175-8239-jbn-2020-0116er

**Published:** 2021-01-22

In the article “The spleen size in patients undergoing hemodialysis”, with DOI code number 10.1590/2175-8239-jbn-2020-0116, published at Brazilian Journal of Nephrology, 2020, on the keywords:

Where it was written:

Polysomnography

Should read:

Sonography

